# High-Depth PRNP Sequencing in Brains With Sporadic Creutzfeldt-Jakob Disease

**DOI:** 10.1212/NXG.0000000000200054

**Published:** 2023-01-19

**Authors:** Alexander G. Murley, Yu Nie, Zoe Golder, Michael John Keogh, Colin Smith, James W. Ironside, Patrick F. Chinnery

**Affiliations:** From the Department of Clinical Neurosciences (A.G.M., Y.N., Z.G., M.J.K., C.), School of Clinical Medicine, University of Cambridge, Cambridge Biomedical Campus; Medical Research Council Mitochondrial Biology Unit (Y.N., Z.G.), University of Cambridge, Cambridge Biomedical Campus; Translational and Clinical Research Institute (M.J.K., P.F.C.), Newcastle University, Newcastle Upon Tyne; and Centre for Clinical Brain Sciences (C.S., J.W.I.), University of Edinburgh, Cambridge, UK.

## Abstract

**Background and Objectives:**

Sporadic Creutzfeldt-Jakob disease (sCJD) has established genetic risk factors, but, in contrast to genetic and acquired CJD, the initial trigger for misfolded prion aggregation and spread is not known. In this study, we tested the hypotheses that pathologic somatic variants in the prion gene *PRNP* are increased in sCJD, potentially leading to the seeding of misfolded prion protein.

**Methods:**

High-depth amplicon-based short read sequencing of the *PRNP* coding region was performed on postmortem brain tissue from patients with a clinical and neuropathologic diagnosis of sCJD (n = 142), Alzheimer disease (AD) (n = 51) and controls with no clinical or neuropathologic diagnosis of a neurodegenerative disease (n = 71). Each DNA sample was sequenced twice, including independent PCR amplification, library preparation, and sequencing. We used RePlow to call somatic variants with high sensitivity and specificity and optimal sequence kernel association test to compare variant burden between groups.

**Results:**

Two sCJD cases had somatic (variant allele frequency 0.5–1%) *PRNP* variants not previously identified, but with high in silico predicated pathogenicity. However, the pathogenicity of these variants is uncertain, as both located in the octapeptide repeat region where no point variations have previously been associated with sCJD. There was no overall difference in burden somatic *PRNP* in sCJD compared with controls and a lower burden compared with Alzheimer disease.

**Discussion:**

Somatic variants in *PRNP* are unlikely to play a major role in sCJD but may contribute to the disease mechanism in a minority of cases.

Creutzfeldt-Jakob disease (CJD) is a rapidly progressive, fatal neurodegenerative disease caused by aggregation and spread of a misfolded prion protein. This pathologic process can result from germline mutations in the prion protein gene *PRNP*^1^ or after iatrogenic^2^ or dietary^3^ exposure to the misfolded prions. However, CJD occurs sporadically in most cases,^4^ and the underlying cause is not known. Defining the mechanism of sporadic CJD (sCJD) may reveal new targets for disease-modifying therapy.

sCJD has known genetic risk factors. Germline *PRNP* polymorphisms can increase or decrease the risk of developing disease,^1^ and de novo *PRNP* mutations occurring at an early stage of embryogenesis have been reported.^5^ Genome-wide association studies have identified other polymorphisms associated with sCJD, including GAL3ST1 and STX6.^6^ Even postmitotic cells such as neurons continue to acquire genomic variants during aging,^7,8^ raising the possibility that somatic *PRNP* mutations could occur in the brain during childhood or adult life. Theoretically, mutations in a small number of cells could result in a seed of protein misfolding that spreads through the brain to cause sCJD^9^

In this study, we aimed to detect somatic *PRNP* variants in brain tissue from patients who died with sCJD and to determine whether any detected variants were likely to cause or modify disease etiology. Our hypotheses were that there would be a higher burden of variants in sCJD compared with controls and that patients with sCJD would have pathologic mutations in brain tissue. To enable accurate detection of low-level somatic variants, we performed PCR amplicon-based high-depth sequencing of the PRNP protein coding region. We replicated the analysis, including PCR amplification and sequencing to control for experimentally induced artifacts. We then used a probabilistic variant caller, RePlow, validated to analyze technical replicates with high sensitivity and specificity.^10^ This showed 2 likely pathogenic variants in 2 sCJD cases, but no overall increase in variant burden in sCJD compared with control groups.

## Methods

DNA extracted from brain tissue from the frontal lobe of patients with a clinical and neuropathologic diagnosis of sCJD (n = 142), Alzheimer disease (AD) (n = 51), and controls with no clinical diagnosis of dementia and no significant neuropathology at postmortem (n = 71) were used in this study. Further details for all participants included in the final analysis are shown in Table 1. Cases with sCJD were collected from the Edinburgh Brain and Tissue Bank, University of Edinburgh, United Kingdom. DNA from control samples, with either a neuropathologic diagnosis of Alzheimer disease or cases without significant neuropathology, was collected from the Medical Research Council (MRC) UK Brain Banks.^11,12^ A positive control with a heterozygous germline E211Q *PRNP* mutation was included in the sequencing pool. This sample was further diluted with DNA from a control with no *PRNP* variant to generate variant allele frequencies (VAF) of 25%, 5%, and 0.5% and enable test of sensitivity. This was repeated twice, so in total, there were 2 positive controls for each VAF per replicate.

**Table 1:**
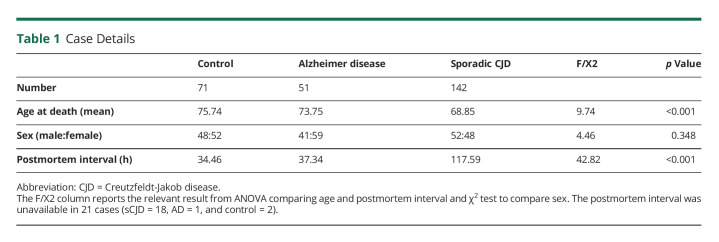
Case Details

The coding sequencing of the *PRNP* gene was amplified using primers and PCR protocol as detailed in reference 13. Samples were indexed using Nextera XT reagents and sequenced with an Illumina MiSeq system (Illumina Inc, San Diego). Each DNA sample was sequenced twice, including independent PCR amplification, library preparation, and sequencing. Sequencing reads were preprocessed following the Genome Analysis Toolkit best practices workflow: data preprocessing for variant discovery.^14^ Samples were mapped to the Genome Reference Consortium Human Build 38. We used the RePlow variant caller to jointly analyze our library-level replicates and to profile and remove background errors.^10^ The background somatic mutation rate was set at 1.75 × 10^−9^, based on modeling from ultra-high-depth sequencing of 173 human brains.^12^ All other model parameters were kept at the default parameters. We used Pindel^15^ to identify large-scale deletions, only reporting deletions called in both replicates.

Variant burden between groups was calculated using the pairwise optimal sequence kernel association test (SKAT-O) in R (version 4.0.3) with sequencing depth, age at death, sex, and postmortem interval as covariates.^16^ Missing postmortem interval values were replaced for the mean group value for this analysis. The resulting *p* values were Benjamini-Hochberg adjusted for multiple comparisons. Samples from 7 cases were not successfully sequenced (mean depth <100) in both replicates and were removed from further analysis.

Variants were annotated using Ensembl.^17^ We followed the American College of Medical Genetics guidelines for the interpretation of sequence variants.^18^ We considered variants to be inherited (germline) if the VAF was 40%–60% or somatic is the VAF was <40%.^19^

### Standard Protocol Approvals, Registrations, and Patient Consents

The study had ethical approval from the local research ethics committee (ID: 13/YH/0310). All participants gave informed consent or, if lacking mental capacity, through a consultee process according to UK law.

### Data Availability

Anonymized data are available on request for academic purposes.

## Results

Parallel amplicon-based sequencing of the *PRNP* protein coding region was performed on the *PRNP* protein coding region at a mean depth of 3,764 (range 1,874–5,398) across all participants. The mean sequencing depth was higher in sCJD (3,908.99) compared with both AD (3,670.12) and controls (3,542.15) (F: 9.9, *p* < 0.001, relevant Benjamini-Hochberg–adjusted *p* values < 0.05). VAF in 2 replicates in the final data set very strongly correlated (R = 0.99, *p* < 0.0001). We confirmed the sensitivity of the RePlow model by successfully detecting all positive controls down to and including a VAF of 0.5%.

First, we looked at the likely germline variants, defined by a VAF of 40%–60%. Two germline variants were detected. The same missense variant (20.4699380G>A) was present in 1 case of sCJD (VAF = 53.6%) and 1 control (VAF = 54.2%). This variant is known to be a benign polymorphism (G54S).^1^ The remaining 2 variants were synonymous and have been previously identified and classified as benign.^20^ One was present in a single control case (20.4699424 T>C, VAF = 50.6%) and the other in a subset of all disease groups (20.4699571 A>G sCJD n = 9, mean VAF = 45.2%, AD n = 4, mean VAF = 43.5%, control n = 3, mean VAF = 44.7%).

**Figure 1:**
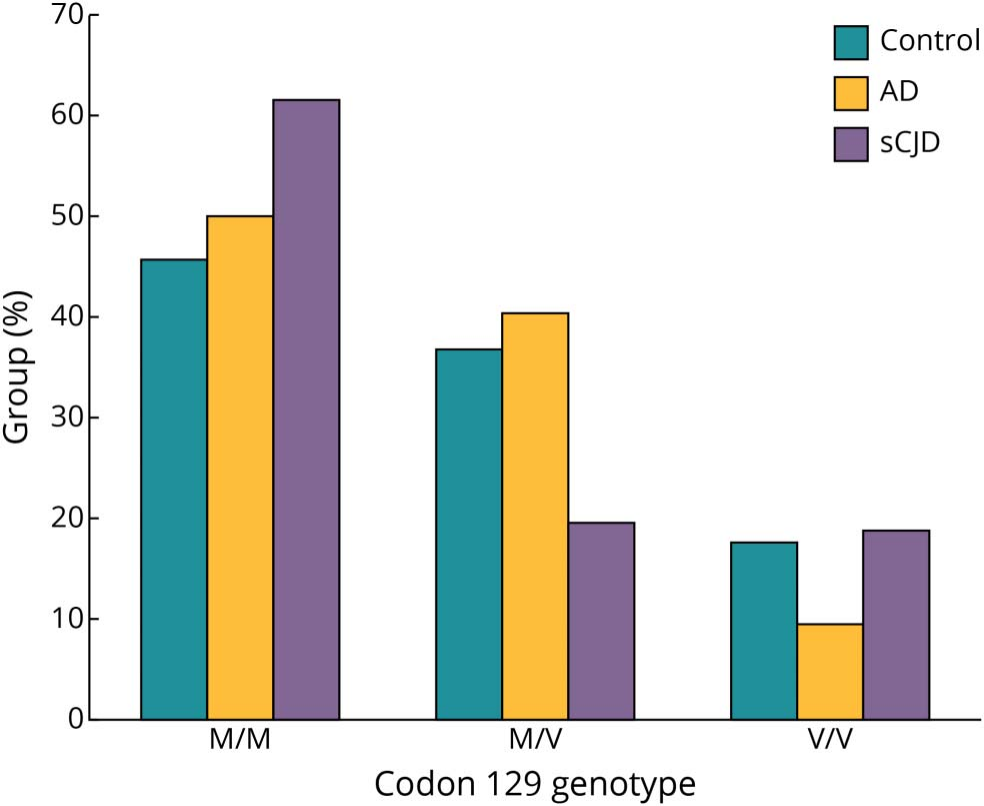
Codon 129 Genotype Barplot of the 3 codon 129 genotypes. AD = Alzheimer disease; M = methionine; sCJD = sporadic Creutzfeldt-Jakob disease; V = valine.

We also compared codon 129 genotype between groups. Most sCJD cases (n = 135) had known codon 129 genotype status in the MRC Brain Bank,^11^ which we confirmed in all cases. Sporadic CJD cases had a significantly higher prevalence of homozygous methionine alleles compared with both Alzheimer disease (F = 9.70, Benjamini-Hochberg–adjusted *p* = 0.001) and unaffected controls (F = 6.93, Benjamini-Hochberg–adjusted *p* = 0.031). Codon 129 genotype did not vary between AD and healthy controls (*p* > 0.05) (Figure 1).

Next, we looked at somatic variants (VAF <40%) in the PRNP coding region across both sequencing runs. Two sCJD cases had a somatic variant (VAF 1% and 0.93%) with high in silico predicted pathogenicity. These were adjacent missense variants within the octapeptide repeat region. Both occurred in codon 74 (GRChg38 positions 20.4699440 and 20.4699441) with an amino acid change from glycine to arginine. These changes are both predicted to change protein function with high PolyPhen and Combined Annotation-Dependent Depletion scores (Table 2). They were not present in any other participants in our study. Integrated Genome Viewer^21^ plots of the aligned reads containing these variants are in eAppendix 1, links.lww.com/NXG/A578.

**Table 2:**
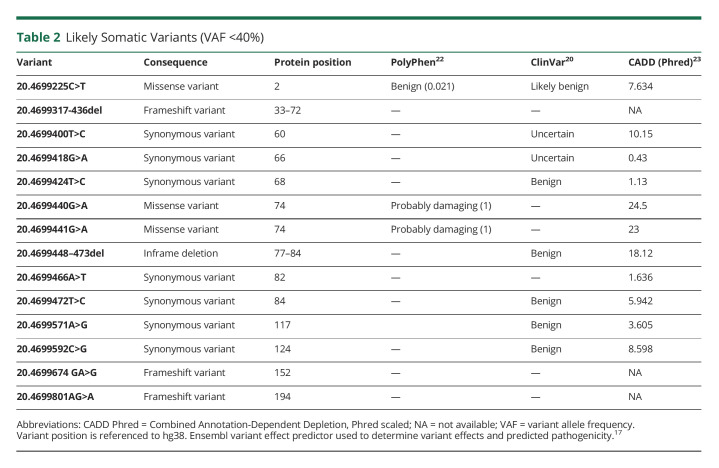
Likely Somatic Variants (VAF <40%)

**Figure 2:**
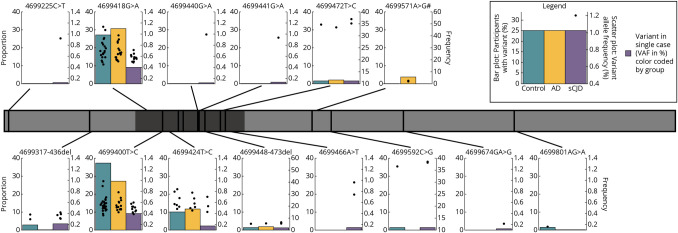
Variants in the *PRNP* Protein Coding Domain The gray bar represents the coding region of the *PRNP* gene. The darker gray section shows the location of the octapeptide repeat region. Black vertical lines refer to the position of called variants. These variants connect to plots, which show, first, the proportion of cases in each group with a variant at this position (bar plot, left y-axis) and, second, the allele frequency of these variants (scatter plot, right y-axis). Note that the scale of the y-axis varies between plots to aid data visualization. Codon 129 is not shown in this figure.

Fifteen other somatic variants across 103 cases were detected in the coding region of the *PRNP* gene (Table 2, Figure 2). There was no difference in the overall somatic variant burden between controls without neurodegenerative disease and sCJD (SKAT-O burden test *p* = 0.33) or AD (*p* = 0.33). There was a trend toward a higher burden of somatic variants in AD compared with sCJD (*p* = 0.031). These results did not change if likely germline variants (VAF >40%) were included in the analysis (SKAT-O burden test, sCJD vs control *p* = 0.58, sCJD vs AD *p* = 0.043, AD vs control *p* = 0.48).

## Discussion

In a large (n = 142) cohort of patients, we identified 2 sCJD cases with likely somatic *PRNP* variants with high in silico predicted pathogenicity. Our methods included ultra-high-depth sequencing, technical replicates, and the validated RePlow variant caller^10^ to minimize the effect of technical artifacts. We included positive controls to confirm a detection threshold of VAF = 0.5%.

Two cases had low-level (VAF ∼1%) variants in *PRNP* that have not previously been described (not present in the gnomAD or ClinVar databases) but have high in silico predicted pathogenicity. The only 2 somatic variants with high predicted pathogenicity were in sCJD cases. There are limitations to using in silico predictions of pathogenicity, which do not always accurately categorize known benign and pathogenic *PRNP* variants in sCJD. No cases of familial CJD have ever been associated with point mutations in the octapeptide region (the location of both variants), which further limits the interpretability of potential pathogenicity of these variants. The overall burden of somatic *PRNP* variants in sCJD brain tissue was also no different to healthy controls, and there was a trend to lower burden in sCJD compared with AD brains. The relevance of the 2 *PRNP* variants in codon 74 is therefore uncertain at present.

The most conservative interpretation of our findings is that somatic *PRNP* mutations are not enriched in sporadic CJD brains, but there are alternative explanations for our findings. Somatic mutations may be present at a level below the sensitivity (VAF ∼0.5%) of our sequencing and bioinformatic pipeline and still affect a sufficient number of cells to seed the spread of prion pathology. Alternatively, any cells containing pathogenic somatic *PRNP* mutations could have died and undergone apoptosis, being among the first cells to be affected by disease. Third, although the frontal lobe typically has a high burden of disease in sCJD, the regional onset of sporadic CJD varies, and it is unlikely that we were sampling the region of disease onset in all individuals.^24^

Our study has other limitations. First, we could not confirm the germline origin of any variants as parental samples were not available (as sCJD is a late-onset disease). Blood samples were also not available for analysis, so we cannot confirm that any somatic variants are restricted to brain tissue. Second, a large proportion of the variants in our results were in the octapeptide repeat region. Abnormal expansions of this region (between 4 and 12) are associated with familial CJD.^1^ PCR amplification and alignment difficulties during analysis of short read sequencing data may increase the probability of technical artifacts.^25^ Recent methods have been developed to measure low-level mosaicism repeat expansion using short read sequencing data.^26^ However, the octapeptide expansion in *PRNP* is technically challenging to measure using these techniques. Third, patients with sCJD had a younger age at death compared with control groups, which is relevant as somatic mutations increase with age.^7^ This could explain why *PRNP* variants were less frequent in sCJD than in the AD brains from older individuals. We included age as a covariate in our burden analysis, but this difference may still confound our results by masking a true increase in burden in sCJD. sCJD cases had a longer postmortem delay, which is unavoidable due to the increased postmortem restrictions and precautions required in prion disease, but is unlikely to have been a significant confounder in our analysis. Finally, PCR amplicon-based sequencing can introduce artifacts that appear as low-level variants.^27^ Our approach significantly reduced this likelihood with PCR and library-level sample replication combined with use of a model specifically validated to call low-level variants in replicated samples.

In conclusion, although we found possibly pathogenic *PRNP* variants in 2 participants with sporadic CJD, overall, there was no increased burden of variants in brain tissue from participants with sCJD compared with controls. Our study has limitations, but we suggest that our largely negative results are definitive, with both a large sample size and robust methods to control for technical artifacts. This suggests that somatic mutations are not the principal cause of prion aggregation in sporadic CJD but may contribute to the etiology of a minority of cases.

## Glossary

AD: Alzheimer disease
CJD: Creutzfeldt-Jakob disease
sCJD: sporadic CJD
SKAT-O: optimal sequence kernel association test
VAF: variant allele frequency
